# Tracking the spatiotemporal evolution of groundwater chemistry in the Quaternary aquifer system of Debrecen area, Hungary: integration of classical and unsupervised learning methods

**DOI:** 10.1007/s11356-025-36175-z

**Published:** 2025-03-01

**Authors:** Musaab A. A. Mohammed, Norbert P. Szabó, Viktória Mikita, Péter Szűcs

**Affiliations:** 1 Faculty of Earth and Environmental Sciences and Engineering, University of Miskolc, Egyetemváros, 3515 Miskolc, Hungary; 2 National Laboratory for Water Science and Water Security, Institute of Water Resources and Environmental Management, University of Miskolc, Miskolc, Hungary; 3 College of Petroleum Geology and Minerals, University of Bahri, Khartoum, Sudan

**Keywords:** Groundwater quality index, Multivariate statistics, Pannonian Basin, Saturation index, Self-organizing maps

## Abstract

Monitoring changes in groundwater quality over time helps identify time-dependent factors influencing water safety and supports the development of effective management strategies. This study investigates the spatiotemporal evolution of groundwater chemistry in the Debrecen area, Hungary, from 2019 to 2024, using indexing, machine learning, and multivariate statistical techniques. These techniques include self-organizing maps (SOM), hierarchical cluster analysis (HCA), principal component analysis (PCA), and groundwater quality indexing (GWQI). The hydrochemical analysis revealed that Ca-Mg-HCO₃ is the dominant water type, with a temporal shift toward Na-HCO₃, reflecting increased salinity driven by ongoing rock-water interactions. SOM analysis showed a transition from heterogeneous to more uniform groundwater chemistry over time, suggesting greater stability in the aquifer system. Elevated salinity zones shifted spatially due to changes in groundwater recharge and flow patterns, while hardness intensified and expanded, indicating continued carbonate dissolution. HCA highlighted temporal shifts in groundwater composition, with six clusters identified in 2019 and five clusters in 2024, reflecting a gradual homogenization of water quality. PCA further confirmed this trend, linking it to underlying hydrochemical processes, such as water–rock interactions, with limited contributions from anthropogenic influences. The GWQI analysis indicated a general improvement in groundwater quality over time, with most regions meeting drinking water standards. However, specific areas exhibited signs of localized contamination, requiring targeted management. These findings underscore the importance of continuous groundwater quality monitoring to detect emerging trends and guide resource management. The study highlights the need for sustainable practices to safeguard water resources and ensure long-term water security in the Debrecen area.

## Introduction

In recent decades, groundwater resources have transformed their chemical composition due to several factors including changes in land use patterns, climate conditions, and anthropogenic activities (Varsányi 1992; Mester et al. 2020; Mohammed et al. 2023; Alizadeh et al. 2024). This transformation has been further complicated by population growth and has led to increased groundwater extraction, particularly in regions where groundwater serves as the primary water supply source (Mohamed et al. 2023). The resulting intensive pumping has given rise to numerous challenges affecting both the quantity and quality of groundwater resources (Su et al. 2020; Ghaffari et al. 2021; Noori et al. 2021). The evolving nature of groundwater systems necessitates a proper understanding of temporal change in groundwater quality to ensure water safety. This includes knowledge of chemical evolution patterns, the nature of water–rock interactions, and their temporal variations (Pillai et al. 2020). These factors are crucial as groundwater interacts with its environment over time, leading to dynamic changes in chemical composition while causing deterioration in water quality. The complexity of these interactions highlights the importance of comparing current and historical hydrochemical data to identify trends and the factors influencing water quality over time (Maghrebi et al. 2021; Ning et al. 2024).

Classical statistical methods often lack the sensitivity and resolution needed to detect subtle variations and interactions within hydrochemical datasets (Patel et al. 2023). To overcome these limitations, modern statistical and machine learning techniques, such as self-organizing maps (SOM), principal component analysis (PCA), and clustering methods, have been widely adopted (Oliveira et al. 2023). Clustering techniques group groundwater samples with similar characteristics, aiding in the identification of distinct water types (Das et al. 2022; Mavaluru et al. 2024). SOM, a neural network-based clustering method, is particularly effective for visualizing high-dimensional data, revealing patterns in groundwater chemistry that may not be immediately apparent (Licen et al. 2023; Zhang et al. 2023). Unlike traditional clustering methods, SOM simultaneously clusters and visualizes data, offering an intuitive representation of complex relationships (Rahman et al. 2022; Alitane et al. 2024). PCA, on the other hand, reduces the dimensionality of datasets while preserving most of the variance. By transforming correlated variables into a set of uncorrelated components, PCA simplifies data interpretation, highlights key influencing factors, and uncovers underlying patterns within the dataset (Abdulsalam et al. 2022; Gan et al. 2023). The integration of these machine learning and statistical methods into groundwater quality assessment provides a robust framework for monitoring and preserving water resources over time (Garba et al. 2023). These techniques enable the identification of emerging trends, the evaluation of influencing factors, and the assessment of potential risks (Mushtaq et al. 2021), ensuring proactive and informed management of groundwater quality.

The water supply system in the Debrecen area relies heavily on groundwater resources extracted from the underlying Quaternary aquifer system in different water fields. The groundwater in waterfields shares a similar origin, as indicated by their comparable hydrochemical characteristics, which suggest recharge from surface sources (Szanyi 2004). Moreover, natural factors and anthropogenic factors, such as the mineral composition of the aquifer rocks, and agricultural activities contribute to the baseline groundwater chemistry (Simon et al. 2023; Carpio 2024). Previous studies have focused on spatial variations in groundwater chemistry with limited attention to temporal changes (Szanyi 2004; Carpio 2024). The dynamic nature of groundwater systems, and evolving environmental pressures, makes the temporal assessment of the groundwater status necessary to identify trends and potential threats (Mester et al. 2024). This study aims to understand groundwater dynamics by conducting a spatiotemporal evaluation of hydrogeochemical changes in the Debrecen region. While groundwater quality in this region is influenced by natural processes and anthropogenic activities, there remains a gap in understanding how these factors contribute to temporal variations in groundwater chemistry. To address this limitation, this research systematically analyzes both historical and current groundwater chemistry data through hydrogeochemical modeling and unsupervised machine learning methods. These methods are highly effective in analyzing complex groundwater datasets, identifying patterns, and distinguishing factors influencing water quality. This comprehensive approach to temporal hydrogeochemical analysis represents an important step forward in understanding urban groundwater systems and their seasonal dynamics and contributes to the development of effective water resource management strategies.

## Study area

**Fig. 1:**
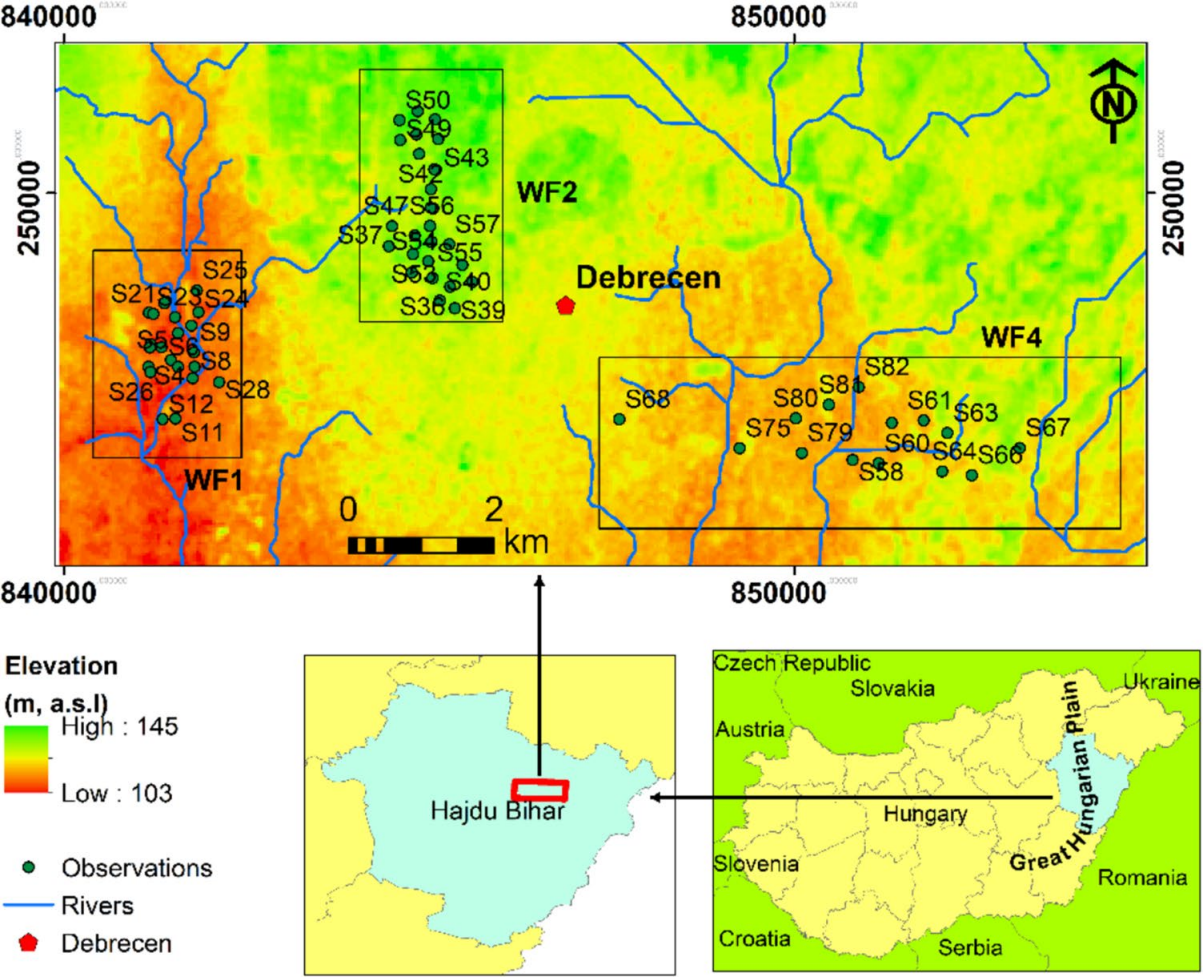
The geographical location of the study area and the distribution of the main groundwater fields (WF1, WF2, and WF4)

The study site extends across 400 km^2^ around Debrecen City within the Hajdu Bihar County, Eastern Hungary (Fig. 1). This area is located in the eastern part of the Great Hungarian Plain (GHP) and is shaped by tectonic movements, erosion, and ongoing sedimentation (Püspöki et al. 2021). These dynamic geological forces have created a diverse topography, with elevations varying from 103 to 145 m above sea level (a.s.l.). Climatologically, the region boasts a predominantly continental climate evidenced by average annual temperatures between 10 and 11° C. Precipitation varies throughout the year, ranging from 550 to 600 mm.

**Fig. 2:**
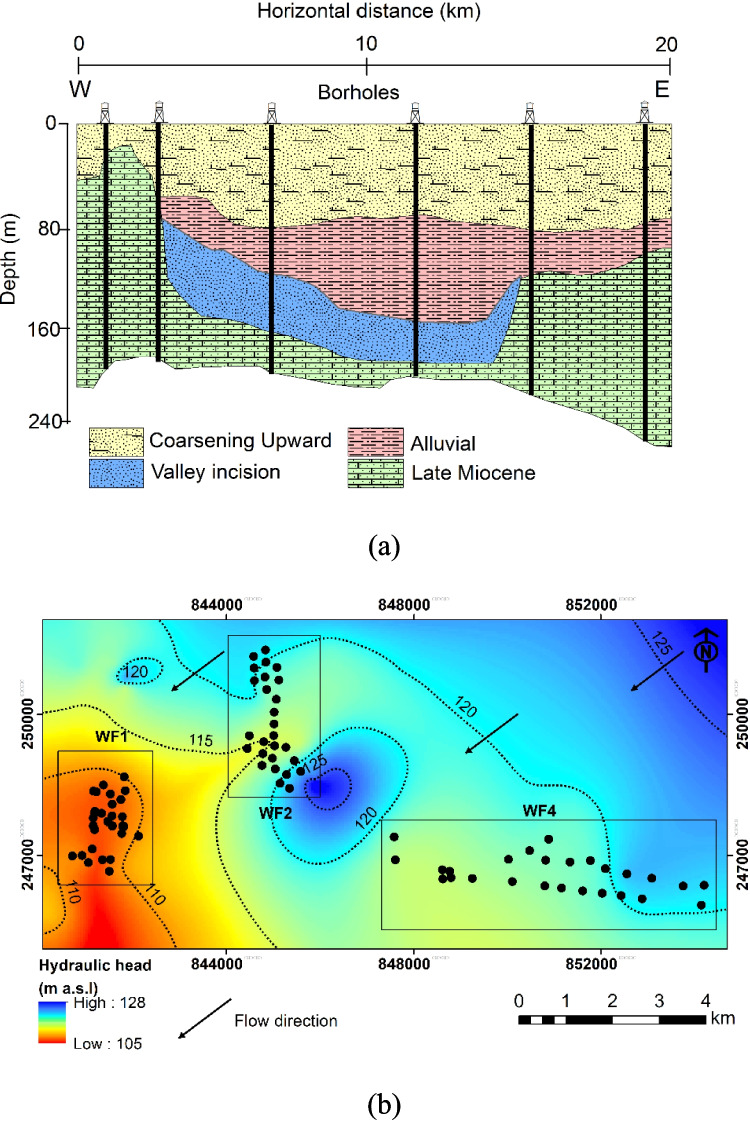
**a** Geometry of the Quaternary aquifer system and the distribution of the main hydrostratigraphical units and **b** groundwater flow in the Quaternary aquifer system

The surface of the study area is dominated by Quaternary deposits composed of fluvial sediments, including river gravels and sandy loess. These deposits can be subdivided into Upper, Middle, and Lower Pleistocene units. The Lower and Upper Pleistocene sub-units comprise river and overbank sediments, while the Middle Pleistocene unit is characterized by fine-grained fluviolacustrine deposits (Püspöki et al. 2013). A recent investigation by Carpio (2024) indicated that the Quaternary sequence in the Debrecen area is divided into three hydrostratigraphic units (Fig. 2a). The first unit, the incised valley unit, resembles an elongated ribbon of sand and gravel, oriented northeast-southwest with minimal clay content. Above this lies the alluvial unit, characterized by a consecutive sand body exhibiting horizontal variability and interspersed with silty clay deposits. Finally, the coarsening upward unit presents a diversity of clay, silt, and sand. The hydraulic conductivity of the main aquifer unit (Incised Valley) varies between 3 to 7 m/d. However, the conductivity within the other units varies considerably from 0.001 to 5.4 m/d. In the GHP, the upper system is governed by the gravity that guides groundwater flow. In this system, precipitation acts as the primary source of groundwater recharge. However, on the regional scale, the study area is located between the recharge zone of Nyírség’s alluvial fans in the northeast and Hortobágy’s discharge area in the southwest (Tóth and Almási 2001). The groundwater flow from the recharge zones in the northeast to the discharge zones in the southwest (Fig. 2b).

## Methods

**Fig. 3:**
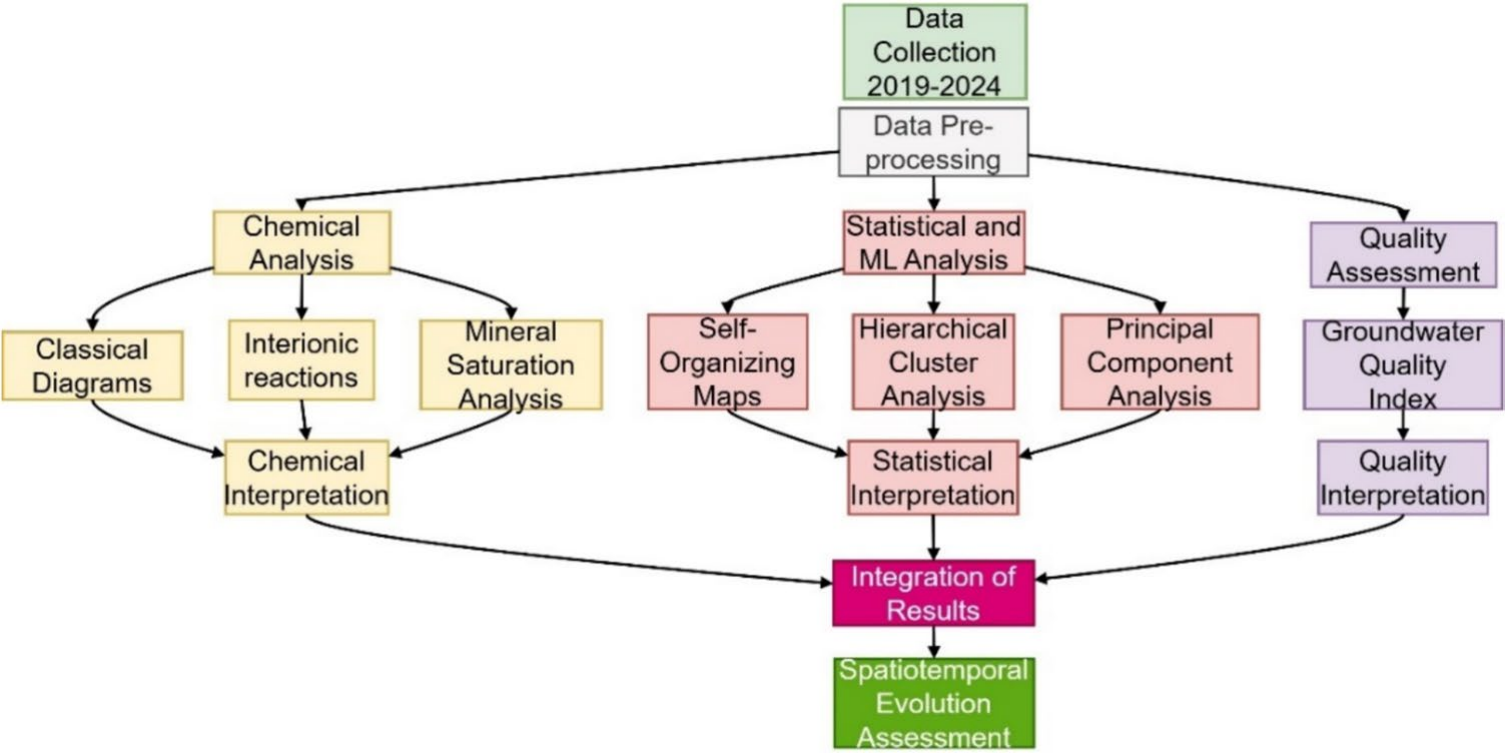
The workflow of the current study to investigate the spatiotemporal evolution of groundwater in the study area

The methodological approach of this study involves a systematic analysis of groundwater quality data collected from 2019 to 2024 in the Debrecen area. The workflow of the study is summarized in Fig. 3. The analysis proceeded along three main pathways: chemical analysis, statistical and machine learning analysis, and quality index assessment. The chemical analysis included classical diagrams, interionic reactions, and mineral saturation analysis. Statistical analysis employed SOM, HCA, and PCA to identify patterns and relationships in the data. GWQI was calculated to assess the overall water quality trends. The results from these methods were then integrated to provide an understanding of the spatiotemporal evolution of groundwater quality in the study area.

### Groundwater sampling

In this study, groundwater samples were collected annually from January 2019 to January 2024 to monitor water quality variations over time in the three groundwater fields (WF1, WF2, and WF4) in the Debrecen area. The choice of annual data for this study was based on the relatively slow variation in groundwater quality compared to surface water quality. Groundwater sourced from deep wells exhibits slower temporal changes due to the extended residence time of water within the subsurface. Most of the groundwater wells (around 91%) are screened in the incised valley hydrostratigraphic units at a depth range between 80 and 160 m. Each sampling round involved a standardized procedure following the Hungarian Standard Methods (MSZ 448/3–47) to ensure consistency in sampling methods and data comparability across years. Water samples were drawn from designated boreholes at consistent depths to capture representative groundwater chemistry without surface contamination. The collected groundwater samples were analyzed for a range of essential chemical parameters, including total dissolved solids (TDS), calcium (Ca), magnesium (Mg), sodium (Na), potassium (K), bicarbonate (HCO₃), chloride (Cl), and sulfate (SO₄). These parameters were selected to provide a comprehensive assessment of water quality and identify any potential shifts in ion concentration. The statistical summary of the analyzed parameters, including their minimum, mean, and maximum values, is presented in Table 1. To ensure the reliability of the analytical results, the quality of each analysis was verified using an electrical balance approach (Appelo and Postma 2005).

**Table 1:** The simple statistics of the analyzed parameters between 2019 and 2024

Parameter (mg/L)	2019			2024			WHO standard
	Mean	Minimum	Maximum	Mean	Minimum	Maximum	
TDS	608.6	270.5	1246.9	611.5	506	911.1	1000
EC	950.9	422.7	1948.3	955.6	790.6	1423.6	1500
Ca	85.9	13	111	86.9	2.2	114	200
Mg	25	3.46	57	18.8	1	24.1	50
Na	29.2	1.76	161	36.8	23.7	263	200
K	1.9	0.1	21.6	1.75	0.8	2.1	12
HCO_3_	453.3	107	1100	461.7	389	771	350
Cl	10.1	5	82	3.3	0	8.6	250
SO_4_	3	0	32	2.1	0	22.6	250

### Hydrochemical diagrams and saturation indices

Hydrochemical diagrams including Piper (1944) and Gibbs (1970) plots are used to identify water types and dominant geochemical processes affecting groundwater quality. The Piper diagram is a trilinear plot that combines two triangular fields and a central diamond field to represent the major cations and anions in groundwater samples. The Gibbs plot is used to examine the relationship between major ion chemistry and potential mechanisms controlling groundwater composition. The Gibbs plot helps to identify the dominant hydrochemical processes influencing groundwater chemistry such as weathering dominance, atmospheric precipitation, or evaporation effects.

The saturation indices (*SI*) calculations are used to assess the saturation state of groundwater to minerals. *SI* is a calculated value used to determine the equilibrium state of groundwater using Eq. 1 as

1 $$SI=log\left(\frac{IAP}{Ksp}\right)$$

where *IAP* is the ion activity product of the dissolved species in water and *Ksp* is the solubility product constant of minerals. An *SI* of zero indicates that the groundwater is at equilibrium for the mineral, meaning there is no net dissolution or precipitation. If *SI* is positive (*SI* > 0), the groundwater is supersaturated, suggesting a tendency for the mineral to precipitate. Conversely, a negative *SI* (*SI* < 0) implies undersaturation, indicating a tendency for the mineral to dissolve.

### Self-organizing maps

The self-organizing map (SOM) is an unsupervised neural network technique that is highly effective for clustering and visualizing high-dimensional data by projecting it onto a lower-dimensional space. In this study, SOM is applied to analyze the relationships between various hydrochemical parameters and their spatiotemporal variations (Belkhiri et al. 2018). The SOM was initially proposed by Kohonen (1982) and operates on the principle of competitive learning. In this application, each neuron in the SOM represents a groundwater sample, characterized by a weight vector (*W*_i_) of the same dimension as the input data *X* = (*x*_1_, *x*_2_, … *x*_n_). The training process involves iteratively presenting hydrochemical data to the network, identifying the best matching unit (BMU) which is the neuron whose weight vector is most like the input data, and updating the weights of the BMU and its neighboring neurons based on Euclidean Distance. The BMU can be found using Eq. 2 as

2 $$\text{BMU}=\Vert X-{W}_{\text{i}}(t)\Vert$$

where $\Vert X-{W}_{\text{i}}(t)\Vert$ represents the Euclidean distance, ${W}_{\text{i}}(t$) is the weight vector of node *i* at time step *t*. The BMU is the neuron with the smallest Euclidean distance to the input vector. Once the BMU is identified, the weight vectors of the BMU and its neighboring nodes are updated to move closer to the input vector.

### Multivariate statistical methods

Multivariate statistical methods, including Pearson correlation analysis, principal component analysis (PCA), and hierarchical cluster analysis (HCA), are used in this study. Pearson correlation analysis was used to examine the linear associations between groundwater quality variables. The correlation coefficients help to determine the strength and direction of relationships among ions, providing an overview of potential interdependencies within the dataset (Mohammed et al. 2022b). PCA was conducted to reduce the dimensionality of the dataset and to identify major factors responsible for variability in groundwater quality. This method transforms the original correlated variables into a smaller set of uncorrelated components, with each principal component representing a combination of the original variables that accounts for a significant portion of the variance (Gan et al. 2023). HCA was applied to classify groundwater samples into clusters with similar chemical characteristics. HCA groups samples based on their proximity in the data space, forming clusters that minimize internal variance while maximizing external separation (Das et al. 2022). The dendrogram generated through HCA visually represented these relationships, allowing for clear identification of groundwater samples with shared hydrochemical features.

### Groundwater quality index

The groundwater quality index (GWQI) is a measure that combines various water quality parameters into a single value representing the overall quality of water. The main steps in calculating the GWQI include selecting the parameters, establishing standards, calculating quality ratings and sub-indices, applying weights, and computing the overall index. In this study, TDS, Na, K, Ca, Mg, HCO₃, Cl, and SO₄ are used. Each parameter has a standard limit (*S*_i_) as set by the World Health Organization (WHO 2011). The quality rating (*Q*_i_) for each parameter is calculated to express the relative concentration of each parameter (Ci) concerning its standard limit. The *Q* is calculated using Eq. 3.

3 $${Q}_{\text{i}}=\left(\frac{{C}_{\text{i}}}{{S}_{\text{i}}}\right)\times 100$$

Each parameter is assigned a weight (Wi) based on its relative importance to overall water quality and potential health impacts (Table 2). In this study, the weight is determined by expert judgment and literature. The sub-index for each parameter can be calculated using Eq. 4.

**Table 2:** The selected parameters for GWQI calculation and their assigned weights

Parameter (mg/L)	WHO Standard (WHO 2011)	Weight	Relative weight
TDS	1000	5	0.2
Ca	200	3	0.12
Mg	50	3	0.12
Na	200	2	0.08
K	12	2	0.08
HCO_3_	350	4	0.16
Cl	250	3	0.12
SO_4_	250	3	0.12

4 $${\text{SI}}_{i}=\text{Wi}*\text{Ri}$$

The sum of the weights (W) for all selected parameters is calculated and the overall GWQI is computed by summing up all the sub-indices and dividing by the total sum of weights. This can be expressed using Eq. 5.

5 $$\text{GWQI}= \frac{\sum {S}_{i}}{W}$$

The GWQI value can be categorized into different classes to interpret water quality more easily. Typical ranges include the following: 0–50, excellent water quality; 51–100, good water quality; 101–200, poor water quality; 201–300, very poor water quality; above 300, unsuitable for drinking or irrigation purposes (Ramakrishniah et al. 2009).

## Results

### Physiochemical parameters

**Fig. 4:**
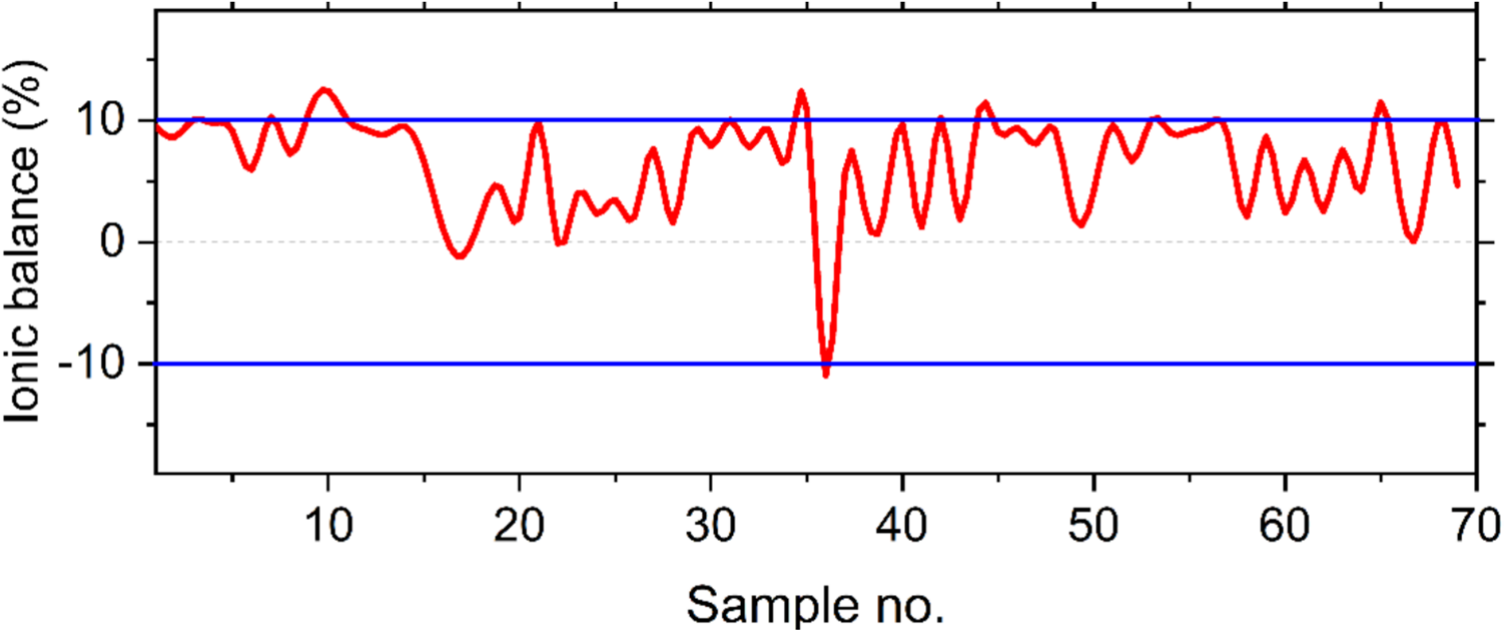
The results of the ionic balance of the groundwater samples

In this study, nine physiochemical characteristics are used to assess the temporal groundwater chemistry in the Debrecen area between 2019 and 2024. The results of the ionic balance analysis of the groundwater samples revealed that most of the samples exhibited an ionic balance within the acceptable range of ± 10% (Fig. 4). A few samples very slightly exceeded this range; however, the deviations were minimal and not significant enough to warrant exclusion. This indicates that the analytical results for the major cations and anions are internally consistent and reliable for further analysis. The descriptive statistic for the analyzed parameters is illustrated in Table 1. TDS in 2019 had a mean of 608.6 mg/L, with values ranging from 270.5 to 1246.9 mg/L. Although the mean TDS was below the WHO limit, some samples exceeded this threshold. In 2024, the mean TDS increased slightly to 611.5 mg/L, but the range narrowed to 506–911.1 mg/L, with all samples falling within the WHO limit. This suggests a modest improvement in TDS levels, with fewer samples exceeding the recommended threshold.

Ca concentrations remained stable across the 2 years, with a mean of 85.9 mg/L in 2019 and 86.9 mg/L in 2024. Throughout the years the ranges fell within the WHO limit of 200 mg/L, indicating consistent Ca levels. Mg levels improved noticeably; the mean concentration decreased from 25 mg/L in 2019, with some samples reaching up to 57 mg/L (slightly above the WHO limit of 50 mg/L), to a mean of 18.8 mg/L in 2024, with all values within safe limits. Na concentration, however, showed an increase. In 2019, the mean sodium concentration was 29.2 mg/L, with a wide range extending up to 161 mg/L. Most values were below the limit, but a few samples exceeded this limit. By 2024, the mean Na level rose to 36.8 mg/L, and the maximum reached 263 mg/L, exceeding standards in some cases. K levels were low, with a mean of 1.9 mg/L in 2019 and a slightly lower mean of 1.75 mg/L in 2024. In 2019, some samples reached 21.6 mg/L, exceeding the standard, but the maximum value in 2024 dropped to 2.1 mg/L, indicating a reduction in K levels across samples.

HCO₃ levels remained high, with a mean of 453.3 mg/L in 2019, ranging from 107 to 1100 mg/L. This exceeded the guidelines for some samples. In 2024, the mean HCO₃ concentration increased slightly to 461.7 mg/L, with a narrower range of 389–771 mg/L, still above the recommended limit in many cases. Cl concentrations were consistently low and well within safe limits. In 2019, Cl had a mean of 10.1 mg/L, with values ranging from 5 to 82 mg/L, far below the WHO limit. By 2024, the mean Cl concentration decreased further to 3.3 mg/L, with a maximum of 8.6 mg/L. SO₄ levels also remained low throughout the years, with a mean of 3 mg/L in 2019 and a mean of 2.1 mg/L in 2024.

### Spatiotemporal variation of chemical characteristics

**Fig. 5:**
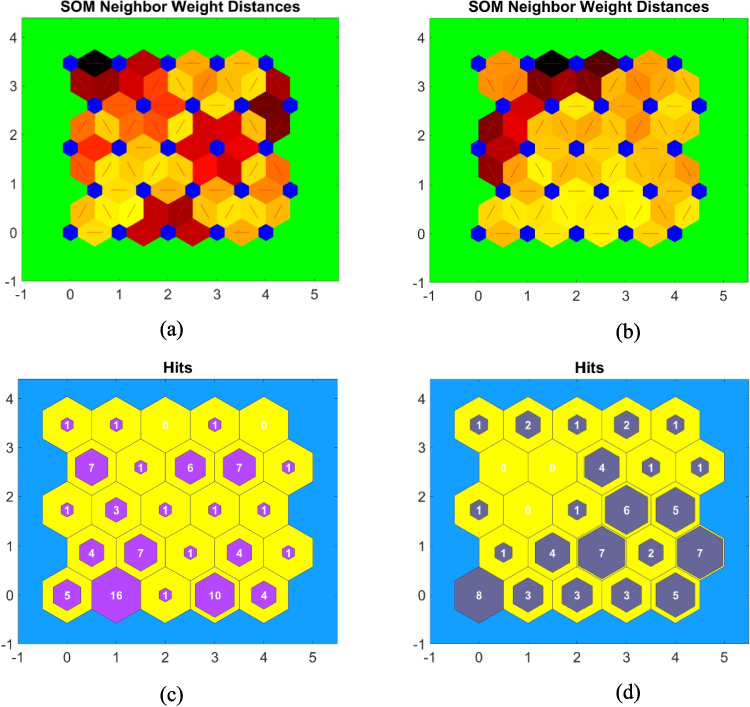
**a**, **b** The neighbor weight distance of SOM. **c**, **d** The hits of SOM showing the distribution of groundwater samples within each neuron for 2019 and 2024, respectively. The dark areas in neighbor weight distance indicate larger distances between neighboring neurons

SOMa of 5 by 5 neurons are used to explore spatiotemporal variations of the physiochemical parameters and groundwater sample in the aquifer system. A hexagonal grid topology was employed, as it provides a more natural representation of neighborhood relationships. The SOM analysis of the samples between 2019 and 2024 showed temporal changes in the distribution patterns. The neighbor weight distances of the 2019 map (Fig. 5a) exhibit clustering patterns with dark red areas indicating larger distances between neighboring neurons, suggesting greater dissimilarity in the data points. In contrast, the 2024 map (Fig. 5b) shows a more gradual transition between neurons which suggests a smoother distribution of chemical properties. The hits maps showed the distribution of samples across the neurons. The 2019 data (Fig. 5c) shows uneven distribution with some neurons capturing up to 16 samples while others remain unused (yellow hexagons). The 2024 hits map (Fig. 5d), however, demonstrates a more balanced distribution with a maximum of 7–8 samples per neuron and fewer empty neurons.

**Fig. 6:**
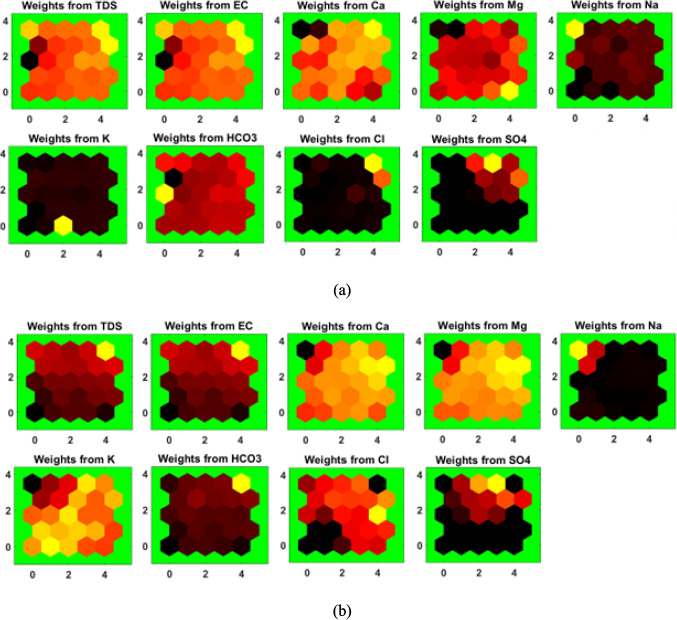
The weight planes of the SOMs for the major chemical parameters for **a** 2019 and **b** 2024

The weight planes of the SOMs from 2019 (Fig. 6a) and 2024 (Fig. 6b) indicated the spatial and temporal distribution of the chemical parameters. The SOMs for TDS and EC throughout the years showed clusters of high values (represented by red colors). These elevated zones indicate areas with increased salinity, which could be attributed to geological formations rich in soluble minerals and localized pollution sources. In 2024, the high TDS and EC areas have shifted slightly, suggesting changes in the spatiotemporal distribution of salinity. In 2019, Ca and Mg concentrations were higher in some clusters. By 2024, these areas of high Ca and Mg have intensified and expanded, pointing to an increase in water hardness over time. In 2019, Na and K show moderate levels across most regions, with some clusters having higher values. By 2024, Na has generally decreased in intensity. K has remained relatively consistent, suggesting stable levels, possibly due to limited sources and lower mobility in the aquifer system. In 2019, regions with high HCO₃ and Cl were evident. By 2024, high concentrations of these anions are still present, with Cl showing slight increases in certain regions. Since HCO_3_ showed similar patterns to that of TDS and EC, it can be indicated that this ion is the most influential parameter to the increased salinity in the study area. SO₄ concentrations in 2019 are generally low. However, in 2024, there is a subtle increase in SO₄ concentrations, though still not widespread.

### Groundwater facies and geochemical processes

**Fig. 7:**
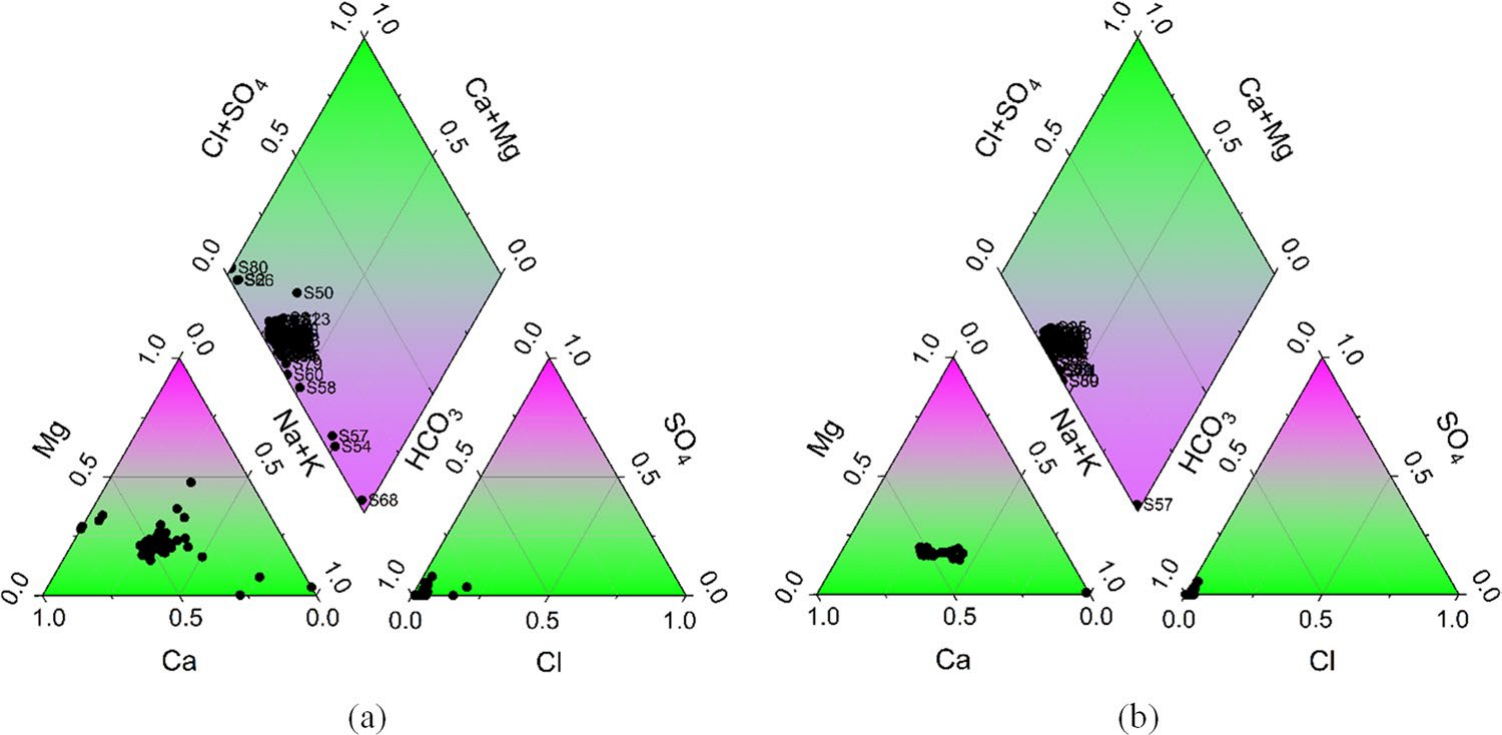
Piper diagrams showing the water types of groundwater samples in **a** 2019 and **b** 2024

The Piper diagram for the groundwater samples in 2019 (Fig. 7a) showed different water facies. The cation triangle shows a strong clustering toward the Ca corner. In the anion triangle, the samples are primarily concentrated around HCO_3_. Most of the samples fall within the Ca-Mg-HCO_3_ region with few samples indicated as Na-HCO_3_ type. These samples of Na-HCO_3_ type are S54, S57 located in WF2, and S68 located in WF4 and associated with maximum concentration of HCO_3_ and Na. On the other hand, samples in WF1 are purely of Ca-Mg-HCO_3_ water type. The Piper diagram for the groundwater samples in 2024 (Fig. 7b) indicated that the overall distribution patterns remain largely consistent with 2019. In the cation triangle, the samples continue to cluster in the Ca-dominant area. Similarly, the anion triangle shows the samples concentrated around the HCO_3_ corner. The diamond field also maintains a predominant Ca-Mg-HCO_3_ water type, with little to no significant shifts observed between 2019 and 2024. Only one sample (S57) remained with a high concentration of Na and HCO_3_.

**Fig. 8:**
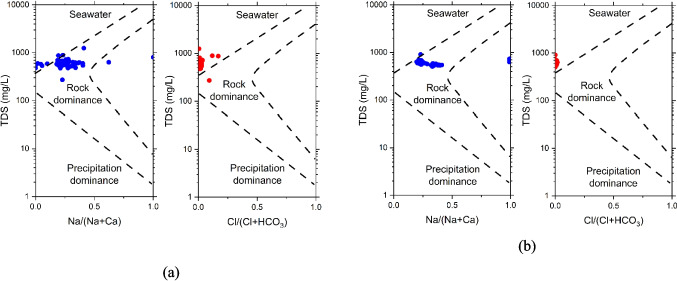
Gibbs plot showing the influencing factor of groundwater samples in **a** 2019 and **b** 2024

The Gibbs plots in Fig. 8a and b identify the dominant processes influencing groundwater chemistry. In 2019, most groundwater samples fell within the “rock dominance” zone for both cation and anion ratios. This suggests that rock-water interaction processes were the primary factor influencing groundwater chemistry. In 2024, the groundwater samples remained in the rock dominance zone. However, there are subtle shifts, with some samples moving closer to the seawater influence zone, particularly in the cation ratio plot. This could suggest an increase in Na levels relative to Ca, potentially due to ongoing rock-water interactions that may introduce Na salts. The samples with high Na levels are in WF4 (S68) where Na-HCO_3_ water types are observed in some locations. There is little change in the anion plot from 2019 to 2024, with samples largely remaining within the rock dominance zone.

### Saturation Indices (SI)

**Fig. 9:**
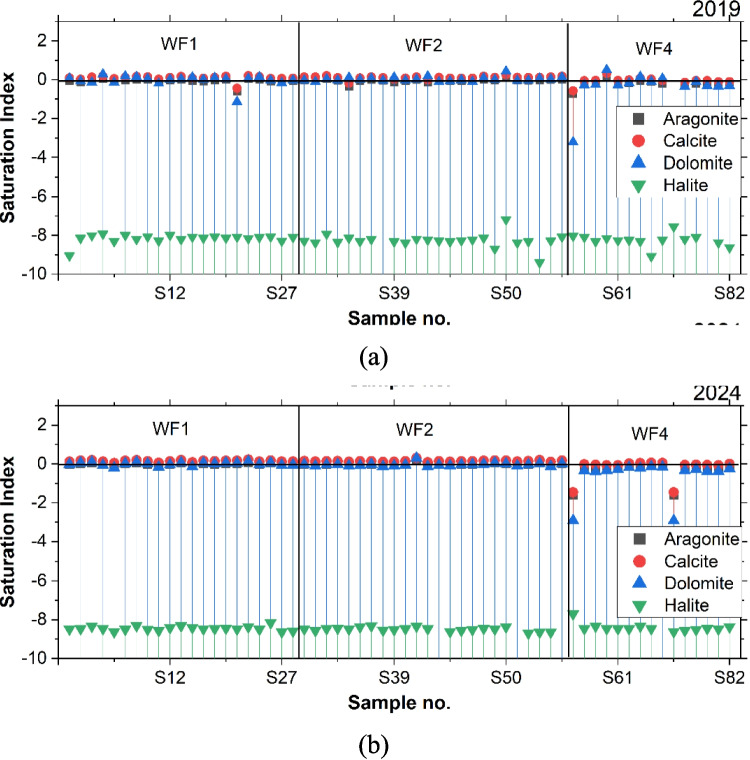
The saturation indices of major minerals in groundwater samples in **a** 2019 and **b** 2024

The SI indicated the hydrogeochemical evolution of the groundwater system across three well fields (Fig. 9a and b). The analysis of carbonate minerals, including calcite, aragonite, and dolomite, demonstrates consistent near-equilibrium and oversaturation conditions with SI values clustered around 0 (± 0.5) throughout the years. However, some outliers are observed, particularly in WF4, where significant undersaturation occurs for these carbonate minerals. The consistency of these patterns between 2019 and 2024 indicates stable carbonate mineral near-equilibrium conditions in the aquifer system. The precipitation of carbonate minerals would reduce HCO₃ concentrations while increasing Ca and Mg concentrations. This is evident since the average concentration of HCO_3_ reduced from 2019 to 2024 and it is likely to continue to reduce in the future.

Halite saturation indices present a different pattern, showing consistent strong undersaturation across all well fields. A slight improvement in SI values is noted from 2019 to 2024. This suggests low Na and Cl concentrations relative to halite solubility and limited influence of evaporite dissolution. WF1 exhibits the most stable SI values throughout the years, with minimal variation in carbonate mineral saturation and consistent halite undersaturation. WF2 demonstrated similar stability, showing slight variations in carbonate mineral saturation while maintaining generally near-equilibrium conditions. WF4 displayed the most variability among the well fields and contains outliers in carbonate mineral saturation, potentially indicating localized different hydrogeological conditions in this part (Carpio 2024).

**Fig. 10:**
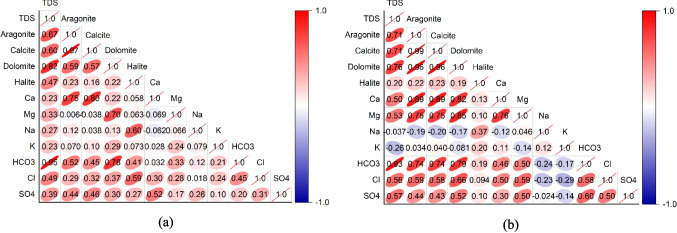
The correlation between the major parameters and saturation indices of the major minerals in groundwater samples in **a** 2019 and **b** 2024

The correlation matrices display the relationships between water quality parameters and SIs for calcite, aragonite, dolomite, and halite. In 2019 (Fig. 10a), TDS exhibited a strong positive correlation with HCO₃ (0.95) and dolomite SI (0.82), indicating that as TDS increases, the likelihood of reaching dolomite saturation also increases. TDS also shows a moderate positive correlation with aragonite SI (0.67) and calcite SI (0.57), suggesting that higher mineral content in groundwater may contribute to the saturation of these minerals. Ca is strongly correlated with calcite (0.80) and aragonite (0.75), consistent with the expectation that Ca-bearing minerals contribute to saturation states. However, there is a lower correlation between TDS and Ca (0.23), indicating that other ions, such as HCO_3_, contribute more to TDS variations. Mg displays weaker correlations overall, which might imply that Mg sources are independent of processes that affect calcium carbonate saturation indices. Na showed low correlations with most SIs. The low correlations between halite SI and other parameters indicate that halite dissolution is not a major factor affecting groundwater composition in 2019.

In 2024 (Fig. 10b), some notable changes in correlation patterns are observed. TDS continues to show a strong correlation with HCO₃ (0.93), and the correlation with dolomite SI remains high (0.76), indicating a continued influence of carbonate mineral dissolution on water composition. However, correlations between TDS and aragonite (0.71) and calcite (0.69) have slightly increased, possibly reflecting an increase in CaCO₃ contributions over time. This shift might indicate that carbonate dissolution or precipitation processes have intensified between 2019 and 2024, potentially due to changes in groundwater recharge (Carpio 2024). There is also a notable increase in correlations between Ca and other parameters, such as TDS, implying a growing influence of Ca-related minerals on groundwater composition. In contrast, Na still shows weak negative correlations with carbonate SIs, supporting the idea that Na remains largely unrelated to the increase of TDS of groundwater.

### Interionic reactions

**Fig. 11:**
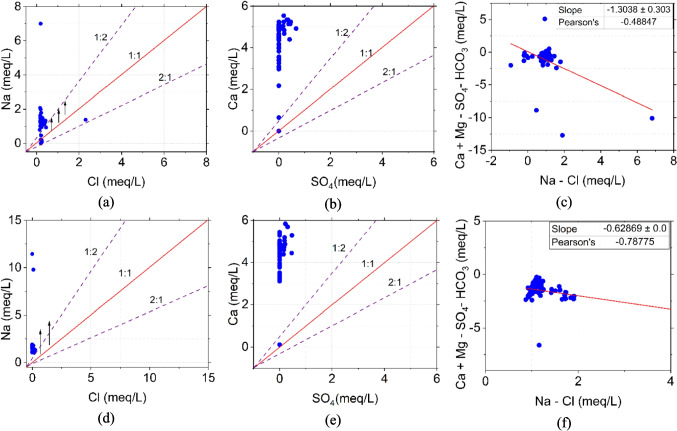
Scatter plots explain the interionic reaction in the aquifer system in **a**–**c** 2019 and **d**–**f** 2024

The interionic relationships in groundwater samples from 2019 and 2024 indicated the hydrogeochemical processes governing the aquifer system. The relationship between Na and Cl (Fig. 11a and d) showed that in 2019, the samples were clustered along and slightly above the 1:1 line, strongly confirming halite dissolution as one of the primary sources of these ions. A single anomalous sample fell below the 1:1 line with a ratio of approximately 1:15, suggesting localized silicate weathering influence (Carpio 2024). By 2024, while maintaining similar distribution patterns, the concentration ranges increased from 12 meq/L compared to the previous 7 mg/L.

The Ca-SO_4_ relationship (Fig. 11b and e) indicated gypsum dissolution processes. The 2019 data showed samples clustering near the 1:2 line (specifically 1.25:2.4), indicating gypsum dissolution as a source of these ions. Some deviation from this ideal ratio suggested additional calcium inputs. The 2024 data maintained similar patterns, though with slightly increased vertical scatter in Ca values. This consistency confirms the stability of gypsum dissolution processes while suggesting the possible intensification of supplementary Ca sources from carbonate weathering and recharge (Szanyi 2004). The ion exchange dynamics are illustrated through the relationship between Ca + Mg − SO_4_ − HCO_3_ and Na − Cl (Fig. 11c and f). The 2019 data exhibited a moderate negative correlation with a slope of − 1.3 and *R*^2^ value of 0.49, indicating active but variable ion exchange processes. By 2024, the correlation showed a slope of − 0.63 and an improved *R*^2^ value of 0.78. This evolution suggests that while ion exchange processes became more consistent (as indicated by the higher *R*^2^ value), they also became less intense as shown by the smaller absolute slope value (Ligavha-Mbelengwa and Gomo 2020).

### Classification of groundwater samples

In this study, HCA is used to classify groundwater samples. Before conducting clustering analysis, the optimal number of clusters is determined to ensure interpretable results. This was achieved using silhouette values to assess how similar each sample to its cluster compared to other clusters (Mohammed et al. 2024). For the 2019 data, the silhouette analysis indicated that six clusters provide the best representation of the groundwater samples. In contrast, the 2024 data was optimally grouped into five clusters.

**Fig. 12:**
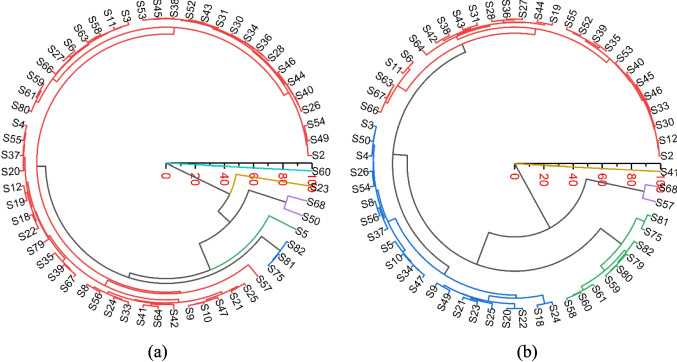
Dendrograms showing the clustering of groundwater samples in **a** 2019 and **b** 2024

The clusters are represented using circular dendrograms (Fig. 12). In 2019 (Fig. 12a), Cluster 1, which comprised the largest group of samples (85%), showed relatively moderate mineralization with TDS ranging from 550 to 650 mg/L and EC values between 840 and 970 µS/cm. This cluster was characterized by moderate Ca (87–105 mg/L), Mg (20–29 mg/L), and Na (24–32 mg/L) concentrations, with HCO_3_ levels ranging from 400 to 460 mg/L and consistently low SO_4_ concentrations. The water type is predominantly Ca–HCO₃. Cluster 2, including samples S75, S81, and S82, exhibited lower mineralization with TDS around 470–520 mg/L and EC around 740–850 µS/cm. This group showed lower Ca (65–70 mg/L) and moderate Mg (20–22 mg/L) concentrations. This cluster also predominantly features Ca–HCO₃ type waters. Cluster 3, represented by sample S5, showed high TDS (732.25 mg/L) and EC (1144.141 µS/cm), with elevated HCO_3_ (549), Ca (94.3 mg/L), Mg (33 mg/L), and Na (36.3 mg/L) concentrations. Cluster 4 (S50, S68) showed very high TDS (848–888 mg/L). S50 is associated with high Ca and HCO_3_, while S68 showed elevated Na and HCO_3_, with reduced levels of Ca. Cluster 5 (S23, WF1) showed very low TDS (270.53 mg/L). The hydrochemical composition of this cluster showed low levels of Ca, Mg, and HCO₃, alongside slight increases in Na and Cl. Cluster 6 (S60, WF4) shows extreme mineralization (TDS, 1246.93 mg/L). This high mineralization is a result of high HCO_3_ and Ca levels.

The cluster analysis of groundwater chemistry data from 2024 revealed 5 distinct clusters (Fig. 12b). Cluster 1, which includes numerous samples (46%) represents a moderate mineralization group with TDS ranging from approximately 580 to 650 mg/L and EC values between 870 and 990 µS/cm. This cluster is characterized by moderate Ca concentrations (80–100 mg/L), relatively low Mg (17–20 mg/L), and Na levels between 24 and 28 mg/L. The HCO_3_ concentrations range from 430 to 470 mg/L, with low SO_4_ concentrations (typically 0–5 mg/L). This cluster appears to represent the typical background groundwater chemistry in the area. Cluster 2, comprising 36% of the samples, shows slightly higher mineralization than Cluster 1, with TDS ranging from 620 to 680 mg/L and EC values between 980 and 1065 µS/cm. This group is distinguished by higher Ca (95–114 mg/L), Mg (20–24 mg/L), and Na (30–32 mg/L) concentrations. The HCO_3_ levels are elevated (470–510 mg/L), and notably, this cluster shows higher SO_4_ (5–15 mg/L) compared to Cluster 1. Cluster 3, which includes 15% of the samples, exhibited the lowest mineralization among all clusters, with TDS ranging from 500 to 550 mg/L and EC values between 790 and 850 µS/cm. This group shows distinct characteristics with lower Ca (62–77 mg/L) and Mg (14–19 mg/L) concentrations but higher Na levels (32–44 mg/L), resulting in a higher Na/Ca ratio compared to other clusters. HCO_3_ concentrations are lower (390–420 mg/L), with minimal SO_4_ concentrations and typical K levels of 1.7–1.9 mg/L.

Cluster 4 consists of only two samples (S57 and S68). These samples show very low Ca (2.2–2.3 mg/L) and Mg (1.0 mg/L) concentrations, coupled with very high Na levels (225–263 mg/L). Despite these extreme variations in major cations, the HCO_3_ levels remain moderate (460–470 mg/L), and K concentrations are notably lower (0.8–1.0 mg/L) than in other clusters. Cluster 5, represented by sample S41, stands out with the highest mineralization of all samples, showing TDS of 911.1 mg/L and EC of 1423.6 µS/cm. This sample is particularly distinguished by its extremely high HCO_3_ concentration (771 mg/L) while maintaining moderate levels of other ions (calcium, 89.4 mg/L; magnesium, 18.7 mg/L; sodium, 26.7 mg/L; potassium, 1.8 mg/L).

**Fig. 13:**
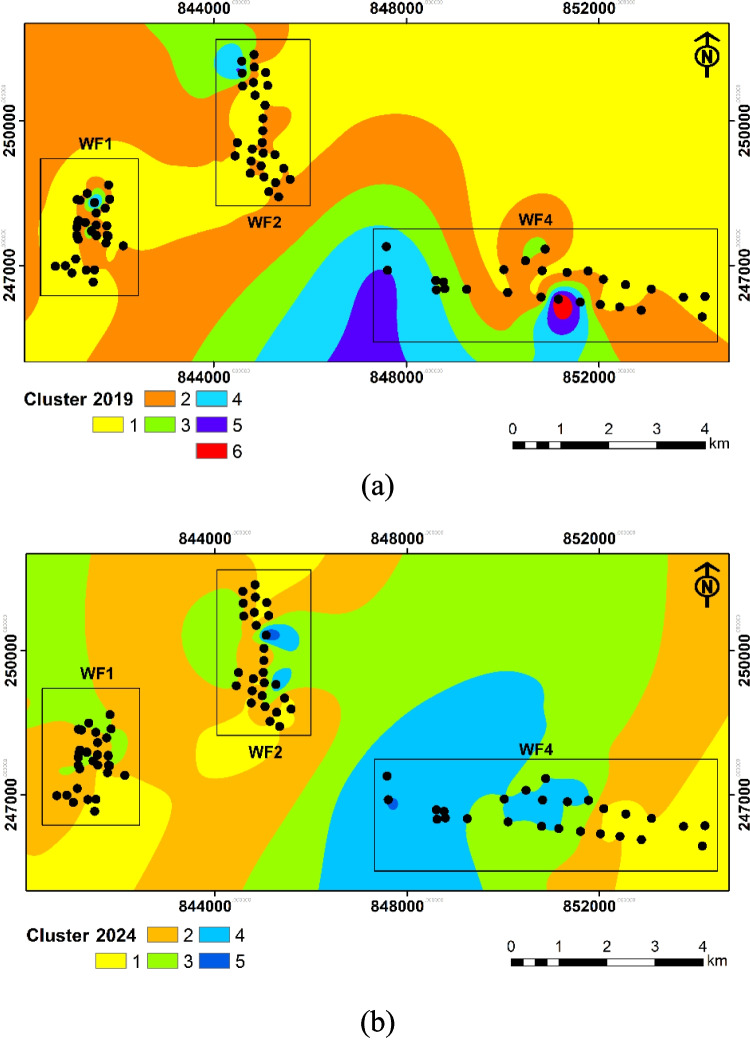
The geographical variation of the extracted clusters within groundwater fields in **a** 2019 and **b** 2024

The spatial distribution of the clusters (Fig. 13a and b) showed changes across different groundwater fields. In WF1, groundwater chemistry remained relatively stable between 2019 and 2024, consistently dominated by clusters representing low to moderate mineralization (Cluster 1 and 2). WF2 shows greater variability and change over the 5 years. In 2019, the groundwater in WF2 was characterized by a mix of moderately mineralized clusters (Cluster 1 and 2) and localized areas of high mineralization (Cluster 3), reflecting spatial heterogeneity and varying residence times. By 2024, there is evidence of consolidation, with more distinct clusters. The presence of highly mineralized (Cluster 5) clusters in 2024 points to ongoing water–rock interaction and potential localized geochemical anomalies. WF4 exhibits the most significant changes between 2019 and 2024. In 2019, WF4 displayed a highly mineralized cluster (Cluster 6). By 2024, the groundwater chemistry in WF4 has further evolved, with a continued dominance of highly mineralized clusters.

### Factor influencing groundwater chemistry

PCA is used to identify the factors influencing groundwater chemistry in the study area. Three PCs explaining 70.1% (2019) and 84.3% (2024) of the total variance are extracted that have eigenvalues greater than one. The PCA revealed temporal changes in the controlling factors between 2019 and 2024 as indicated by the factor loading patterns (Table 3). In 2019, PC1 explains the largest portion of variance with dominant positive loadings from TDS (0.62) and HCO_3_ (0.59), indicating that these parameters were the primary controlling factors in groundwater chemistry. Na also showed moderate positive loading (0.31), while Ca demonstrated slight negative loading (− 0.10), suggesting an inverse relationship with the dominant process (Wu et al. 2021). PC2 in 2019 was characterized by strong positive loadings from Ca (0.47), SO_4_ (0.46), Mg (0.39), and Cl (0.41), representing a secondary mineralization component likely associated with both carbonate dissolution and possible anthropogenic influences (Bouteraa et al. 2019). PC3 showed high loadings from Ca (0.418), SO_4_ (0.416), and Na (0.355), possibly related to mixed ion processes. By 2024, the loading patterns underwent reorganization, with PC1 showing strong positive loadings from Ca (0.524) and Mg (0.511), suggesting an increased dominance of carbonate mineral dissolution processes. PC2 in 2024 displayed high loadings from TDS (0.591) and HCO_3_ (0.502), indicating a shift in the importance of total mineralization to a secondary controlling factor. The emergence of stronger loadings for K (0.421) and HCO_3_ (0.511) in PC3 for 2024 suggests the development of new hydrochemical processes, possibly related to silicate weathering (Gomo et al. 2013).

**Table 3:** The loadings of the extracted principal components in 2019 and 2024

Parameter (mg/L)	2019			2024		
	PC1	PC2	PC3	PC1	PC2	PC3
TDS	**0.62**	0.002	− 0.068	0.06	**0.59**	0.31
Ca	− 0.10	**0.47**	**0.41**	**0.52**	− 0.02	0.001
Mg	0.08	**0.39**	− 0.56	**0.51**	− 0.08	− 0.09
Na	0.31	− 0.42	**0.35**	− 0.47	**0.24**	− 0.05
K	− 0.05	**0.18**	− 0.39	0.29	− 0.35	**0.42**
HCO_3_	**0.59**	− 0.10	− 0.19	0.16	**0.50**	0.51
Cl	0.29	**0.41**	0.07	0.29	**0.34**	− 0.35
SO_4_	0.21	**0.46**	**0.41**	0.19	**0.28**	− 0.57

The bold values means high loading and thus high influence on the PC

**Fig. 14:**
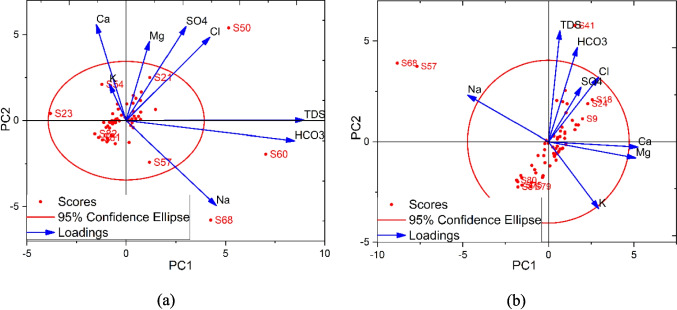
Biplots showing the distribution of the factor scores and factor loadings in **a** 2019 and **b** 2024

The biplots (Fig. 14) provided a visual representation of evolving hydrochemical relationships and sample distributions between 2019 and 2024. The 2019 biplot (Fig. 14a) shows most samples cluster within the 95% confidence ellipse, indicating relatively homogeneous hydrochemical conditions for most of the samples. Notable outliers including samples S50, S60, and S68 demonstrate distinct hydrochemical characteristics, with S60 strongly influenced by TDS and HCO_3_, while S50 shows a strong association with Cl and SO_4_ concentrations. The vector arrangement showed a clear separation between carbonate-related parameters (Ca, Mg) and salinity-related parameters (Na, Cl). In contrast, the 2024 biplot (Fig. 14b) demonstrated a marked evolution in hydrochemical patterns, with a more dispersed sample distribution and modified vector alignments. Samples S68, S57, and S41 appear as significant outliers, indicating localized areas of distinct water chemistry.

### Suitability of groundwater for human consumption

**Fig. 15:**
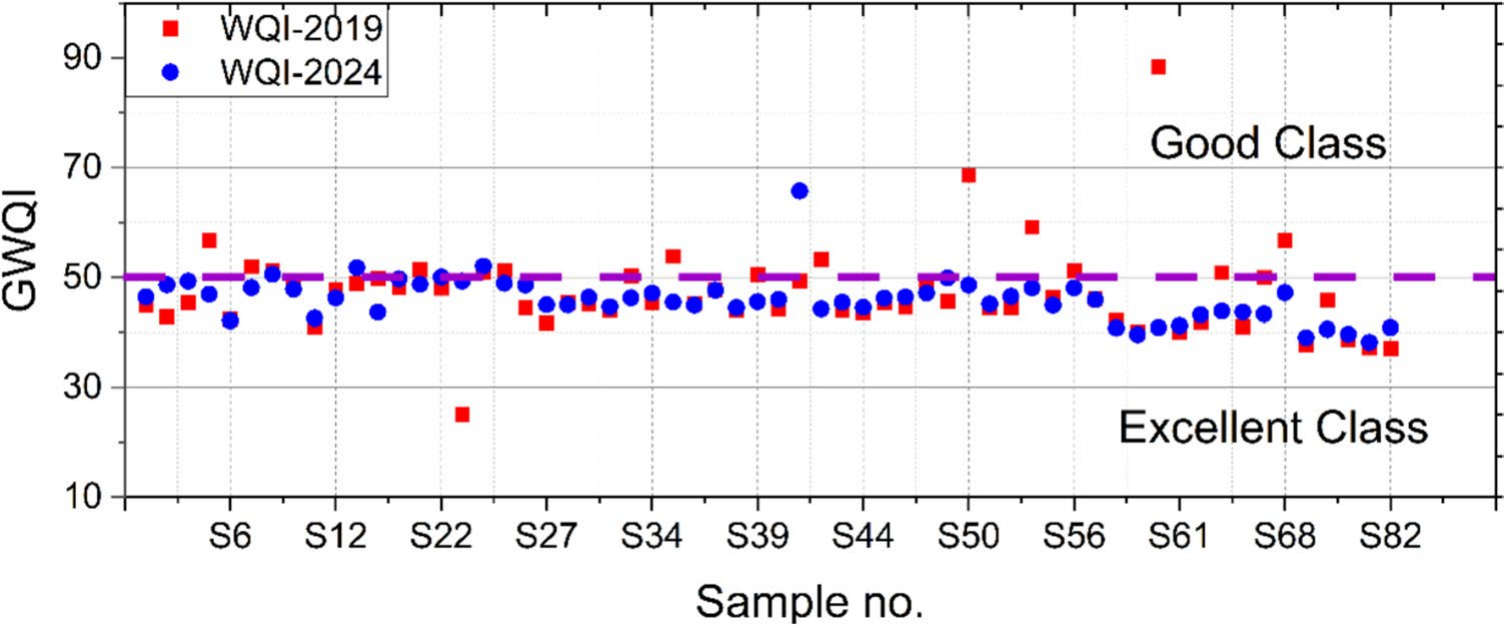
The classification of the groundwater samples based on their calculated GWQI

The suitability of groundwater for drinking purposes is studied with GWQI. The calculated indices revealed an overall improvement in groundwater suitability from 2019 to 2024, with a significant number of samples falling within the excellent class (Fig. 15). In 2019, most of the GWQI values for the samples fall within the range of the good class. However, some outliers have higher GWQI values, reflecting slightly degraded water quality in certain samples, due to localized contamination. In contrast, the GWQI values for 2024 showed a slightly lower distribution, with more samples falling within the excellent class. This may confirm natural purification processes through groundwater recharge (Varsányi 1992).

**Fig. 16:**
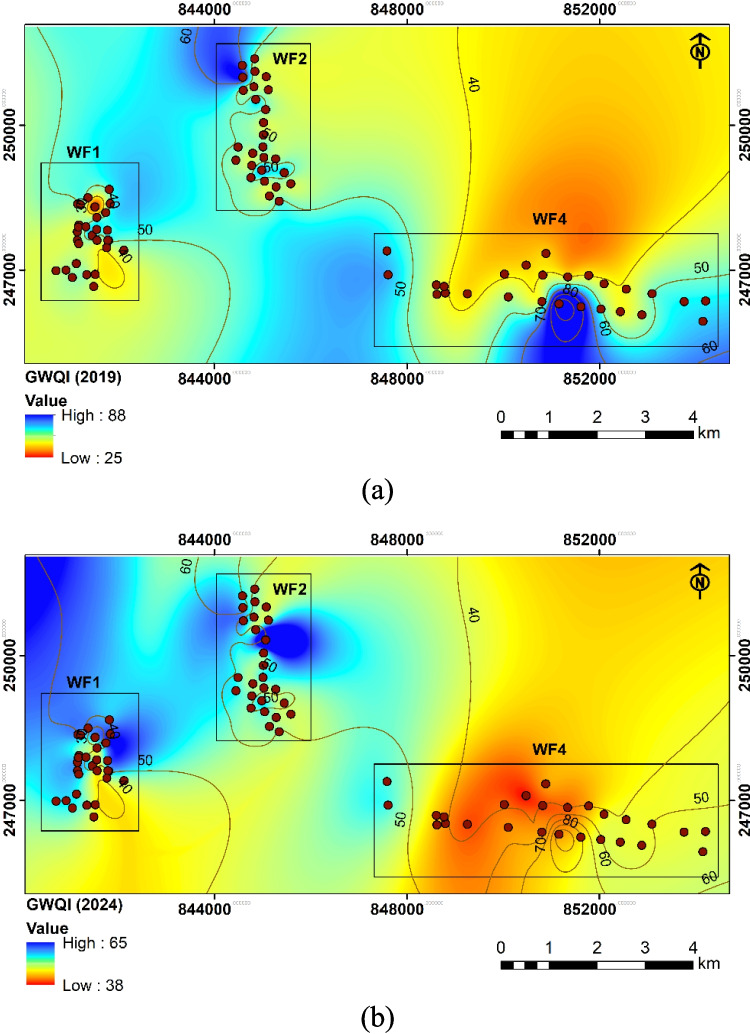
The spatial distribution of the calculated GWQI across the groundwater fields in **a** 2019 and **b** 2024

The distribution of GWQI for 2019 and 2024 across the groundwater fields WF1, WF2, and WF4 revealed spatiotemporal changes in water quality across these regions. In 2019 (Fig. 16a), the GWQI values show a wide range. WF1 displayed a concentration of higher GWQI values around the central part. Similarly, WF2 shows variable GWQI values, with a mix of high and moderate-quality zones scattered throughout, while WF4 exhibited the most heterogeneous GWQI distribution. While pockets of low GWQI values are present, large areas with higher GWQI values suggest more degraded water quality due to increased mineralization in this zone. In 2024 (Fig. 16b), there is a noticeable shift toward generally improved groundwater quality across all fields. The highest GWQI values in WF1 have decreased, and this region shows a larger expanse of low GWQI values compared to 2019. WF2 also exhibits a reduction in high GWQI values, suggesting an overall improvement. In WF4, the high GWQI has diminished, though some regions still exhibit elevated values.

## Discussion

The spatiotemporal variations in groundwater quality in the Debrecen area were investigated in this study using graphical methods, SOM, HCA, PCA, and GWQI. A key finding of this research is the temporal shift in groundwater chemistry toward hydrochemical homogenization between 2019 and 2024, as evidenced by changes in hydrochemical facies, clustering patterns, and the factors influencing groundwater chemistry evolution. The transition from a Ca-Mg-HCO₃ to a Na-HCO₃ water type suggests an increase in salinity, primarily driven by ongoing rock-water interactions. The dominance of the Ca-Mg-HCO₃ water type indicates generally good groundwater quality, although some hardness-related characteristics may be present (Maghrebi et al. 2021). According to Carpio (2024), the presence of the Ca-Mg-HCO₃ water type results from carbonate rock patches within the incised valley unit. More broadly, in the Pannonian Basin, groundwater-type transitions along the flow path from Ca-Mg-HCO₃ to Na-HCO₃ due to ion exchange processes (Varsányi et al. 2011).

The consolidation of hydrochemical clusters, as identified through SOM and HCA from 2019 to 2024, indicated simplified flow paths and modified water–rock interactions. This simplification was accompanied by a narrowing of mineralization ranges and the disappearance of extremely high TDS values (> 1000 mg/L), suggesting an overall improvement in groundwater quality. These findings align with several recent studies while also highlighting the unique characteristics of the study area (Szanyi 2004; Simon et al. 2023; Carpio 2024). The observed spatial heterogeneity in TDS distributions corroborates the findings of Simon et al. (2023) in a similar hydrogeological setting. However, in contrast to their study, which reported persistent TDS hotspots, a transition toward more homogeneous distributions was observed, suggesting the influence of different hydrogeological processes or management interventions.

A significant limitation of our study relates to the deterministic nature of SOM and HCA analysis. The hard clustering approach employed does not account for the inherent uncertainties in hydrochemical data, potentially leading to the oversimplification of complex patterns (Mohammadrezapour et al. 2020). On the other hand, the application of fuzzy SOM and HCA approaches could better represent the transitional nature of hydrochemical processes by allowing samples to belong to multiple clusters with varying degrees of membership (Golaki et al. 2024). Recent studies by Lee et al. (2019) and Lu and Lo (2002) have demonstrated the advantages of fuzzy SOM and clustering in handling hydrochemical uncertainties, resulting in more nuanced classifications that better capture transitional zones. Future research should explore the application of fuzzy SOM to determine whether it provides more robust insights into boundary conditions and transitional hydrochemical states. Another limitation concerns our sampling strategy.

The GWQI analysis revealed overall improvement in water quality despite localized anomalies, suggesting that current management approaches are generally effective. A key limitation in the GWQI calculation is the subjectivity associated with the weighting process, as we relied on expert judgment to assign weights to individual water quality parameters (Gao et al. 2020). While expert-based weighting is a widely accepted approach, it introduces potential biases and uncertainties, as different experts may prioritize parameters differently based on their experience and regional hydrogeological knowledge (Srivastava et al. 2024). To address this limitation, alternative weighting methods could be explored to improve objectivity and robustness in the GWQI calculation. One potential approach is the use of PCA or factor analysis (FA), which derives weights based on the intrinsic variance and correlation structure of the water quality dataset (Abdelaziz et al. 2020; Mohammed et al. 2022a; Zhang et al. 2024). These data-driven approaches reduce human bias by assigning higher weights to parameters with greater influence on overall water quality.

The outcomes of the recent research have several practical applications for groundwater management in the study area and beyond. The identified spatial patterns of water quality parameters provide a foundation for developing targeted extraction regulations, particularly in regions like WF4 that exhibit persistent quality concerns. Similarly, the temporal evolution patterns can inform adaptive management strategies that respond to changing hydrochemical conditions. While this study focused primarily on hydrochemical aspects, groundwater quantity plays a crucial role in the observed patterns and processes. Changes in extraction rates or recharge volumes can significantly influence flow dynamics, residence times, and consequently, water–rock interactions and resulting chemistry (Kamra et al. 2002). Future research should integrate quantitative hydrological data with hydrochemical analyses to develop more comprehensive management models.

## Conclusion

This study evaluated the groundwater quality in the main water fields (WF1, WF2, and WF4) of the Debrecen area, Hungary, over the period 2019–2024. The study employed an integrated approach that combines hydrochemical analysis, self-organizing maps (SOM), hierarchical cluster analysis (HCA), principal component analysis (PCA), and the groundwater quality index (GWQI). The results indicated that the groundwater in the study area is predominantly characterized by the Ca–HCO₃ facies, driven by carbonate mineral dissolution. The temporal analysis highlighted a trend toward more uniform groundwater chemistry, with changes in salinity patterns attributed to variations in recharge and flow dynamics. The GWQI analysis indicated that most samples, especially in WF1, exhibit good water quality suitable for drinking purposes. However, WF4 showed localized contamination associated with the geological conditions and chemical reactions along the groundwater flow paths.

While this study provides critical insights into the spatiotemporal evolution of groundwater quality in the Debrecen area, it is limited by the absence of continuous evaluation of all parameters over the study period. Such an approach could offer a more detailed understanding of temporal dynamics and emerging trends in water quality. Furthermore, while the methods applied effectively identify dominant hydrogeochemical processes, they could be enhanced by incorporating advanced tools, such as isotopic tracing, to better identify recharge sources, contamination pathways, and groundwater flow dynamics. Future research should prioritize continuous monitoring of key contaminants, including nitrate and heavy metals, to address potential human health risks and ensure proactive management of groundwater resources. The findings of this integrated approach contribute to ensuring safe and sustainable access to clean water for domestic use and provide a robust framework for improving water resource management in similar hydrogeological contexts.

## Data Availability

The data used in this research is confidential.
